# Effect of Environmental Exposure on the Pore Structure and Transport Properties of Carbon Nanotube-Modified Mortars

**DOI:** 10.3390/ma13204543

**Published:** 2020-10-13

**Authors:** Panagiota Alafogianni, Konstantinos Dassios, Christos D. Tsakiroglou, Theodore E. Matikas, Nektaria-Marianthi Barkoula

**Affiliations:** 1 Department of Materials Science and Engineering, University of Ioannina, 45110 Ioannina, Greece; pan.alafogianni@gmail.com (P.A.); kdassios@chemeng.upatras.gr (K.D.); matikas@uoi.gr (T.E.M.); 2 Department of Chemical Engineering, University of Patras, 26504 Patras, Greece; 3 Institute of Chemical Engineering Sciences, Foundation for Research and Technology-Hellas, Stadiou Street, Platani, 26504 Patras, Greece; ctsakir@iceht.forth.gr

**Keywords:** pore structure, water absorption, gas permeability, freeze-thaw, sulfate attack, carbon nanotube, mortars

## Abstract

Τhe present study investigates the pore structure and transport properties of carbon nanotube-modified cementitious mortars after exposure to freeze-thaw cycles and immersion to sulfate ion solution (sulfate attack) and compares them to those of un-exposed mortars. The effect of parameters related to carbon nanotube content (within the range of 0.2–0.8 wt.%) and type of dispersant (superplasticizer/surfactant) are investigated. It is found that carbon nanotube inclusion results, overall, in a significant drop of the total porosity before exposure. Results demonstrate that environmental exposure leads to a reduction of the fraction of small diameter pores and a respective increase in capillary porosity for both dispersive agents compared to un-exposed specimens. Diffusion coefficients of nano-modified specimens are lower compared to those of un-modified mortars, both before exposure and after sulfate attack. In the case of freeze-thaw cycling, the diffusion coefficients were found to be higher in carbon nanotube-modified mortars when surfactants were used as dispersants, although with improved gas permeability values.

## 1. Introduction

Cement-based structures are prone to a deterioration of their physical and mechanical properties, which affects long-term performance during their operational lifetime. It is well documented that various forms of environmental exposure (e.g., freeze-thaw, sulfate attack, acid attack, chloride penetration, etc.) may result in a significant loss of functionality or excessive and unpredictable maintenance in cementitious materials. For instance, depending on exposure temperature, pore interconnection, and saturation state of the material, the repeated action of freeze-thaw (F-T) cycles may result in the formation of micro-cracks which, in turn, leads to permanent damage [1,2,3,4,5]. Sulfate attack (S-A) is another common type of environmental exposure that causes swelling, fragmentation, and cracking of the cementitious matrix through the reaction of the calcium hydroxide and calcium aluminate hydrate with sulfate ions and the generation of gypsum and ettringite [6,7,8,9]. Changes in the pore structure along with cracking are linked to alterations in the permeability and transport properties of environmentally exposed cementitious materials, as previously documented [10,11,12]. Zhang et al. [10] studied the moisture absorption and chloride penetration in various types of concrete after F-T cycles and noticed a large increase in the capillary absorption coefficient of conventional concrete, a small increase for frost-resistant concrete, while in the case of concrete with an air-entraining agent, moisture absorption remained almost constant after F-T exposure. In addition, an increase in chloride content and penetration depth with the increase of F-T cycles has been reported for conventional concrete [10]. Wieczorek et al. demonstrated an exponential increase of oxygen permeability over freezing cycles in different types of mortars while moisture absorption results were less sensitive to exposure [11]. At the same time, the study by Jo et al. [12] reported lower permeability in hydrogen-rich water-based mortar presented after exposure in sulfate solutions, due to a denser microstructure.

The imperative need to improve the performance of conventional cementitious materials and to impart multi-functionality and smartness in structures has stimulated great scientific interest towards the use of carbon-based nano-inclusions in cementitious materials [13,14,15,16,17]. Many studies, based on microstructural analysis, suggest that carbon nanotubes (CNTs) contribute to the refinement of the pore network as well as bridging of micro-cracks [18,19,20,21,22,23]. More specifically, in the work of Parveen et al. [18], Nochaiya and Chaipanich [19], and Ghahari et al. [20] authors reported a drop in both porosity and total surface area in CNT-modified cement-based materials. This has been attributed to the filling of mesopores and the creation of a denser microstructure due to a good interaction between the hydration products and CNTs. This finding is also supported by the work of Guan et al. [21], which demonstrated a formation of a compact microstructure after the addition of CNTs. A reduction in porosity ranging between 25% and 47%, also linked with the growth of hydration products into the voids and inter-hydrate spaces of cementitious matrix, has been demonstrated in [22] for CNTs and CNTs-graphene oxide cementitious nanocomposites. Furthermore, in [21,23] the study of the fracture surface of CNT-modified cementitious materials revealed a crack-bridging effect of the nano-phase. This effect leads to crack reduction and enhancement of strength and fracture energy. Contrary to the previous works, Hu et al. [24] reported an increase in the porosity of mortars with up to 0.5% CNTs, linked with the air-entraining action of the dispersant used to facilitate the nano-modification.

Limited research is however available on the transport properties and permeability of these materials after environmental exposure. In this direction, the research by Salemi et al. [25] observed an increase in the water absorption of concrete after 300 F-T cycles, however this increase was considerably lower in samples containing nano-alumina and nano-silica particles. Another study on carbon nano-fibre modified cementitious pastes linked the resistance to sulfate attack with the initial pore refinement as well as the beneficial role of nano-fibres in limiting the crack formation and extension of the tested specimens [9].

Although, as aforementioned, nano-reinforcement is generally believed to have a positive impact on the durability of cementitious materials, it is also clear that the effect greatly depends on the dispersion state of the nano-fillers. One conventional way to introduce CNTs in cementitious matrices is the use of an appropriate dispersant (surfactant or superplasticizer) [18,26,27,28]. Nonetheless, the presence of dispersants is known to interfere with the hardening process and alter the physical properties of the cementitious matrix [26,28]. To this end, we have recently published two studies on the durability of CNT-modified cementitious composites. One study was related to the pore structure and transport properties before environmental exposure [29] and the other related to the structural properties and damage detection capability after the environmental exposure of such materials [30]. Key parameters of interest in both studies was the effect of the amount of CNTs in combination with the type and amount of dispersant used to facilitate dispersion of the nano-filler. Both studies concluded that, apart from the refinement due to the inclusion of CNTs, the type and amount of dispersant also controlled the pore structure and in turn the transport and fracture properties of CNT-modified cementitious materials.

Since environmental exposure is inevitable in constructions, the current study focuses, as the rational next step, on the investigation of the pore structural and transport properties of CNT-modified mortars after environmental exposure. This is of particular interest, since, as already discussed, microstructural changes occur as part of a dynamic process that take place during exposure. So far, most studies attempt to predict the transport properties of nano-modified mortars based on their microstructural characteristics before exposure. However, the initial rate of ingress of harmful substances (i.e., before exposure) is not necessarily conclusive for the durability of a structure after exposure. Next to that, alterations in the processes that prevail during environmental exposure may occur depending on the physical and/or chemical nature of the dispersants of CNTs (surfactants and superplasticizers). For instance, the air entraining action of surfactants could have a positive impact on F-T, while dispersants may interfere with the reaction of the calcium hydroxide and calcium aluminate hydrate with sulfate ions during S-A. Thus, the scope of the current study is to investigate the role of nano-modification on the transport properties of mortars that have been already exposed to F-T and S-A environments. All experiments are conducted on cementitious mortars instead of concrete. The reason behind this selection is that nano-modification is expected to influence the microstructure of the cementitious paste and not to interact with the aggregates. Furthermore, the use of coarse aggregate could negatively influence the microstructure and transport properties with the introduction of discontinuities at the interface between the aggregates and the cementitious paste, and this is another reason that mortars are selected as model systems. For this purpose, specimens with CNT contents ranging from 0.2 to 0.8 wt.%. were prepared using two dispersants, one belonging to the superplasticizer family and the other to the surfactant one. F-T and S-A exposure were selected on the basis that they result in significant microstructural changes and crack formation, and hence are expected to have a significant impact on the transport properties of the nano-modified cementitious materials. The pore structure was evaluated via mercury intrusion porosimetry while the transport properties were experimentally studied through water absorption and gas permeability measurements.

## 2. Materials and Methods

### 2.1. Materials and Specimen’s Preparation

Cementitious mortars were prepared using Ordinary Portland cement ASTM type I 42.5R, natural sand with 2.36 mm maximum diameter, specific gravity of 2.50 kg/dm^3^ and water absorption of 2.44% and tap water. Nano-modification was achieved using multiwalled CNTs (ONEX MW1000C1, Glonatech SA, Athens, Greece), and two dispersive media, namely an anionic surfactant (Sodium Dodecyl Benzene Sulfonate, Sigma-Aldrich, Taufkirchen, Germany) and a poly-carboxylate-based superplasticizer (Viscocrete Ultra 300, Sika Hellas SA., Krioneri, Greece). CNTs were selected based on their geometrical characteristics (length > 10 μm, diameter range 20–45 nm, and specific surface area >150 m^2^g^−1^ [29]) and carbon purity (carbon purity >94%). Sodium-dodecyl-benzene sulfonate was used due to its high efficiency in CNTs’ separation, while poly-carboxylate-based superplasticizers (e.g., Viscocrete Ultra 300) have been previously applied in suspensions specifically targeted for nano-modified cementitious materials owing to their anionic nature and compatibility with cement [29,30]. An anti-foaming agent (TBP) (Tributyl-Phosphate, Sigma-Aldrich, Taufkirchen, Germany) was applied in surfactant-based nano-modified mortars to maintain the air content values constant at 5.5 ± 0.2% [29], while an additional superplasticizer (Viscocrete ultra 600, Sika Hellas SA., Krioneri, Greece) was used in all mixes to keep the workability at 16.3 ± 0.2 cm [29].

Un-modified and CNT-modified mortars were prepared following BS EN 196-1 [31], with constant w/c ratio of 0.5. Three specimens per composition were manufactured using 450 g of cement, 225 g of water and 1350 g of sand. The quantities of all admixtures used for the preparation of the specimens are listed in Table 1 for each composition. Nano-modification was introduced through the water requirement of the mixture, by preparation of aqueous suspensions of CNTs at variable concentrations. For this purpose, CNT/dispersive agent/water suspensions were sonicated according to an optimized protocol that can be found in our earlier works [29,30]. As already discussed, the dispersant/CNT ratio was selected based on the dispersive ability of each agent and its potential side effects on the physical properties of the prepared mortars [29,30]. Thus, the surfactant/CNT ratio was set at 0.5 while the respective superplasticizer/CNT ratio was kept at 1.5. The amount of CNT varied between 0 and 0.8 wt.% of cements in increments of 0.2%, to obtain nano-modified mortars below and above the electrical percolation threshold, which is normally found at app. 0.5–0.6 wt.% for this type of material. Further details about the production of the CNTs suspensions, proportions of materials, and mixing procedure can be found in our previous works [29,30]. The fresh mortars were placed in steel molds for 24 h. After demolding, they were immersed in water baths for another 27 days.

### 2.2. Environmental Exposure and Characterization

**Figure 1:**
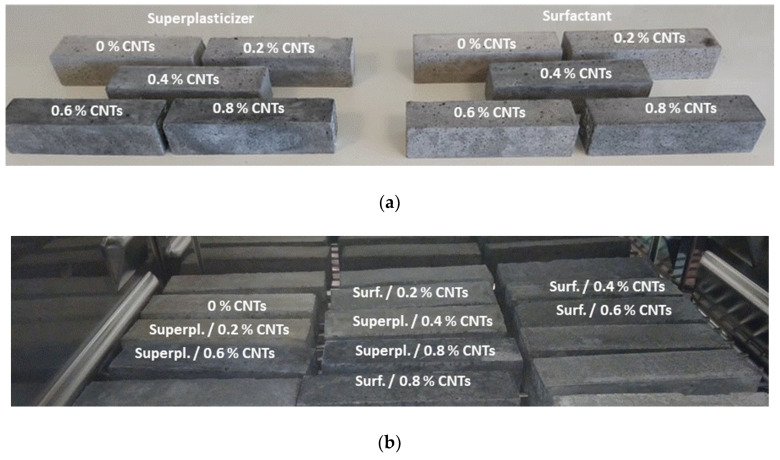

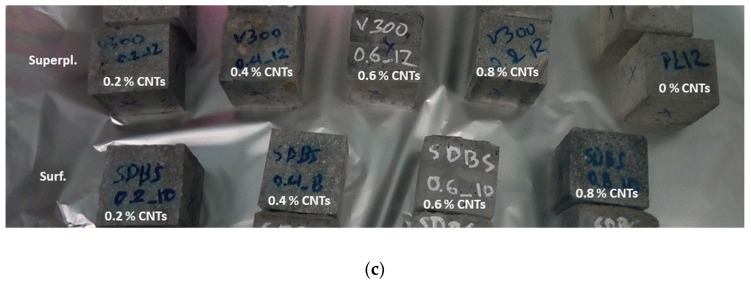
Pictures of un-modified and CNT-modified specimens (**a**) before, (**b**) after F-T and (**c**) after S-A exposure.

Prismatic specimens (40 × 40 × 160 mm^3^) were exposed to 300 successive F-T cycles based on ASTM C666 [32] in a Vötsch VC 4018 environmental chamber (Vötsch Industrietechnik GmbH, Balingen, Germany). During the freezing state, fully saturated specimens were conditioned for 4 h at −18 °C, while the thawing stage included the exposure of the specimens for 4 h at 4 °C and RH~98%. For the S-A exposure, cured, un-exposed specimens (40 × 40 × 50 mm^3^) were immersed in 5% sodium sulfate solution for 16 weeks according to the method described in ASTM C 1012 [33]. Every four weeks, the sulfate solution was replaced with a fresh one. Pictures of the specimens before exposure, after F-T, and after S-A are included in Figure 1.

To define the effect of environmental exposure on the durability of the plain and nano-modified mortars, microstructural characterization, water absorption and gas permeability measurements were performed before and after F-T and S-A.

Microstructural characterization was conducted using a Quantachrome PoreMaster 60 Hg Porosimeter with a measuring capacity between 0.0036–13 μm in mercury/vacuum contact angle of 40°. The pore data were obtained using 3 small pieces (app. 1.5 g) for each composition and exposure state, taken from different areas of crashed specimens (debris obtained after the compression tests-not shown here). Before the mercury intrusion porosimetry (MIP) measurements all specimens were oven-dried at 90 °C for 3 days. More details about the experimental protocol of such measurements can be found elsewhere [29].

**Figure 2:**
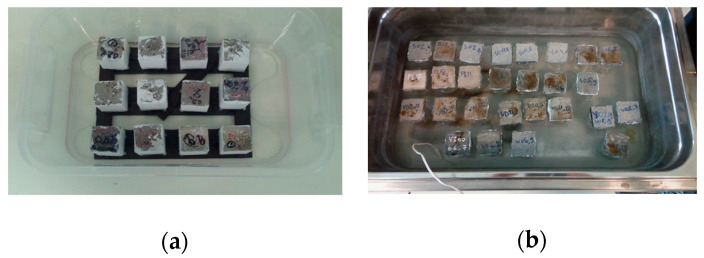
Pictures of the (**a**) water absorption, and (**b**) gas permeability test set-ups.

For the water absorption test, three rectangular specimens (40 × 40 × 50 mm^3^) were immersed in a water container filled with distilled water at ambient temperature (23 °C) (see Figure 2a). Before immersion, all specimens were conditioned at 50 ± 2 °C and RH of 80 ± 3% for 3 days using the VC 4018 environmental chamber and sealed sideways by applying plastic paint to ensure uniaxial diffusion of water. Their mass was defined using an analytical balance (±0.001 mg) (Sartorius Lab Instruments GmbH, Goettingen, Germany).

The relative weight gain of the specimens was calculated using Equation (1):

ΔW_i_ = (W_i_ − W_0_)/W_0_ (1)

where ΔW_i_ is the relative weight gain of the specimens, W_0_ is the weight of the specimens in the dry state, in g, and Wi is the weight of the specimens at specific time intervals up to saturation (app. 320 h), in g.

Since the water absorption profiles obey Fick’s law, the water diffusion coefficient is calculated based on Equation (2):

D_m_= (s^2^πd^2^)/(16ΔW_s_^2^) (2)

where, D_m_ is the water diffusion coefficient, in mm^2^/s, s is the slope of the absorption curves, in %/s^1/2^, d is the thickness of the specimen, in mm, and ΔW_s_ is the relative weight gain at saturation, in %.

The gas vapor permeability was studied by measuring methanol loss through 40 × 40 × 10 mm^3^ specimens using Ashlamsi and Imran’s test set-up [34]. Three specimens per tested condition were initially oven-dried at 90 °C until a constant mass was reached (app. after 3 days) and then placed on top of methanol-containing glasses. The glass-specimen contact area and the sides of the specimens were sealed to ensure unidirectional flow of methanol. All set-ups were weighted to record their initial mass (W_g0_) and immersed up to half of their height in a water bath with controlled temperature (50 ± 3 °C) (see Figure 2b). Weight measurements were conducted during exposure for the determination of methanol’s loss based on Equation (3):

ΔW_g_ = W_g0_ − W_gi_ (3)

where ΔW_g_ is the mass loss of methanol, in g, W_g0_ is the initial mass of the test set-up, in g, and W_gi_ is the mass of the test set-up at specific time intervals, in g

Finally, the mass flow rate of methanol, is calculated as the slope of the linear part of the ΔW_g_ = f(t) curve.

Statistical calculations (mean values and standard deviation) were performed on the water absorption and gas permeability results.

## 3. Results

### 3.1. Pore Structure

**Figure 3:**
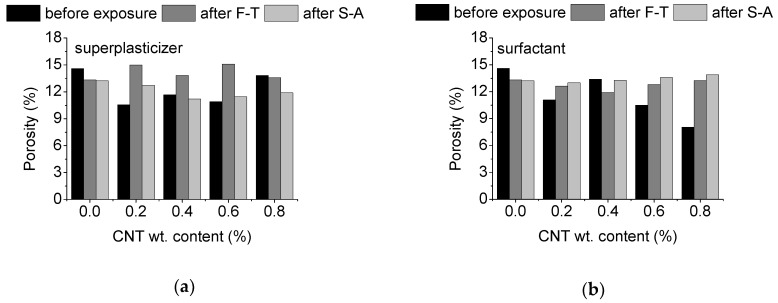
Effect of CNT content and exposure state on the porosity of mortars prepared using: (**a**) superplasticizer, and (**b**) surfactant as dispersant.

Given the fact that the durability of cementitious materials is widely connected to their pore structure, an investigation of the effect of porosity in the mortars was pursued first. Figure 3 depicts the total porosity (%) of the mortar as a function of exposure state for CNT-modified specimens prepared using (a) superplasticizer and (b) surfactant as dispersive agents, respectively. Results from un-modified mortars are also added for reference and comparison purposes. It is observed that the inclusion of CNTs results overall in a significant drop in the total porosity of un-exposed specimens (up to app. 65%). This is more obvious in specimens using surfactant as dispersant and has been linked to the higher ability of pore refinement of well dispersed CNTs [29]. The dispersion of CNTs using superplasticizer is less efficient, thus pore refinement competes with pore formation around agglomerates that are more dominant at high CNT levels [29]. Environmental exposure of the un-modified specimens, results in a small reduction of the total porosity by app. 9%. This can be linked with a continuation of the hydration process during exposure. The beneficial role of nano-modification on the porosity of the prepared specimens is however not maintained after environmental exposure. As observed, most nano-modified specimens show higher porosity values after F-T, compared to their un-exposed state. More specifically, specimens prepared with superplasticizer as dispersant present an overall significant increase, up to 40%, of total porosity after F-T, while similar trends (increase up to 65%) are observed in surfactant-aided specimens. Porosity values after S-A are observed to depend greatly on CNT concentration and dispersive medium. Nano-modified specimens prepared with superplasticizer show an increase in their porosity (app. 20%) at 0.2 wt.% CNTs after S-A, while as the CNT/dispersant content ratio rises, the porosity values approach those of the un-exposed state. Finally, at 0.8 wt.% the porosity drops by app. 15%. In surfactant-based nano-modified specimens, the porosity is higher after S-A, and this is more obvious at high levels of CNTs (up to 70% increase at 0.8 wt.% CNTs, compared to the un-exposed state). Interestingly, the porosity of nano-modified mortars after S-A is similar or slightly lower compared to the respective values of the un-modified specimens after exposure. The same holds for the surfactant-based specimens after F-T. This is not the case in superplasticizer based-mortars after F-T, where porosity values are higher after nano-modification. This can be linked with very high quantities of superplasticizer used for the dispersion of CNTs, which result in high compaction [29], and thus reduced resistance to F-T.

**Figure 4:**
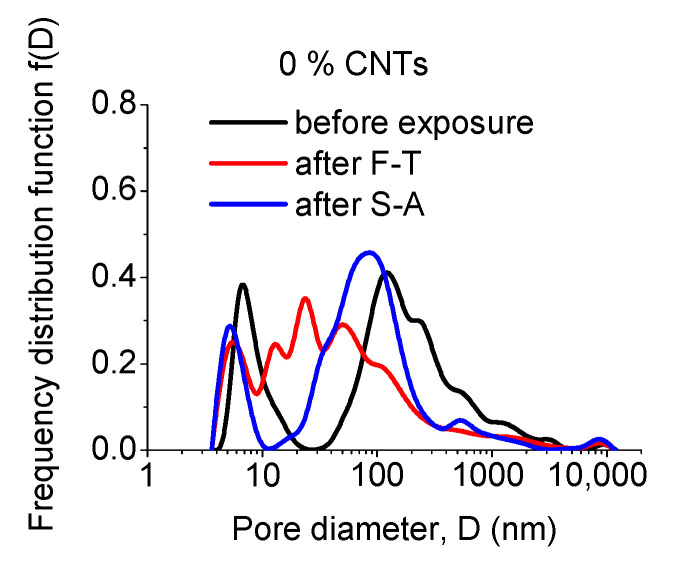
Effect of exposure state on the pore size distribution of un-modified mortars.

**Figure 5:**
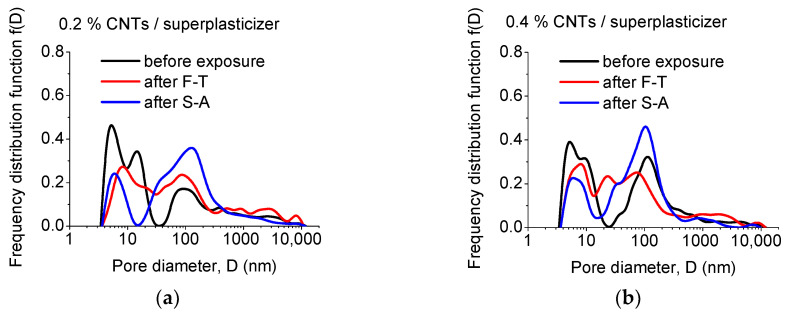

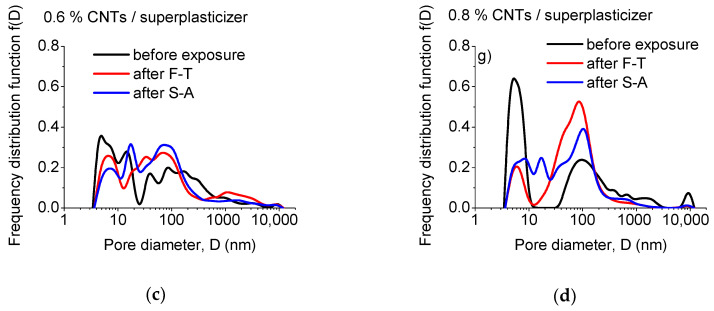
Effect of exposure state on the pore size distribution of mortars with: (**a**) 0.2, (**b**) 0.4, (**c**) 0.6, and (**d**) 0.8 wt.% CNT contents, prepared using superplasticizer as dispersant.

**Figure 6:**
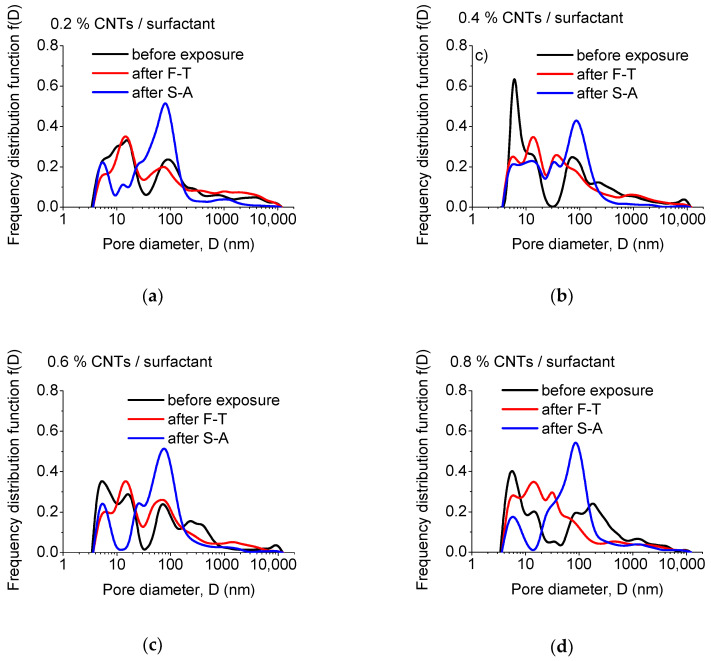
Effect of exposure state on the pore size distribution of mortars with: (**a**) 0.2, (**b**) 0.4, (**c**) 0.6, and (**d**) 0.8 wt.% CNT contents, prepared using surfactant as dispersant.

Although total porosity has been considered as indicative of the durability of cementitious materials, more appropriate measures for such evaluation are the actual pore sizes developed during manufacturing/curing and after exposure. The relevant results, i.e., the pore size distribution of un-modified and CNT-modified mortars prepared with superplasticizer and surfactant as dispersants are presented in Figure 4, Figure 5 and Figure 6, respectively.

As observed in Figure 4, un-modified specimens exhibit pores that are distributed around two separate areas; the first is located around the peak at 7 nm and the latter at 128 nm. F-T and S-A exposures reduce the intensity of the first peak and slightly shift it to lower sizes. New intermediate peaks between 10 and 100 nm are created after F-T, while the second peak is shifted to lower sizes and becomes more dominant after S-A.

The effect of nano-modification becomes apparent by observation of pore size distributions of mortars modified with different CNT concentrations, prepared with the aid of superplasticizer (Figure 5). Prior to exposure, the presence of nano-modification is linked to a clear split of the 7 nm peak, especially for CNT contents up to 0.6 wt.%, as depicted in Figure 5a–c. The peak becomes very well-defined for 0.8 wt.% CNT content (Figure 5d). The peak’s position has been slightly shifted, from 7 to 5 nm, while the split peak/shoulder position can be found at 10–13 nm in the case of nano-modified specimens. Nano-modification leads also in a shift of the peak originally located at 128 nm to 78–114 nm, depending on the CNT content. As in the case of the un-modified specimens, F-T and S-A exposure results in alteration of the microstructure of the CNT/superplasticizer mortars. In most cases, the pore size distribution data demonstrate an increase of the intensity of the peak at app. 100 nm and a respective decrease of the pore population at lower pore sizes, indicating a shift towards larger pores.

Common to superplasticizer-based nano-modified specimens (Figure 5), surfactant-based specimens (Figure 6) also accommodate a considerable amount of their pore population under specific areas located around 10 nm and 100 nm. Again, peaks and shoulders can be found at app. 5 nm and within 13.5–16 nm, depending on the exact composition of the specimens. The peak originally at 128 nm in um-modified specimens, is shifted to 69–91 nm after nano-modification with the aid of surfactant. Furthermore, a shoulder/peak appears at significantly higher pore sizes (180–250 nm) after dispersing CNTs with surfactants. As observed in Figure 6, environmental exposure has a significant effect on the microstructure since it diminishes the pore population around 180–250 nm in all surfactant-based nano-modified specimens. Moreover, the pore population located under the 100 nm peak is significantly reduced and this peak is shifted to lower sizes after F-T. On the contrary, S-A enhances the pores located under the 100 nm region.

**Figure 7:**
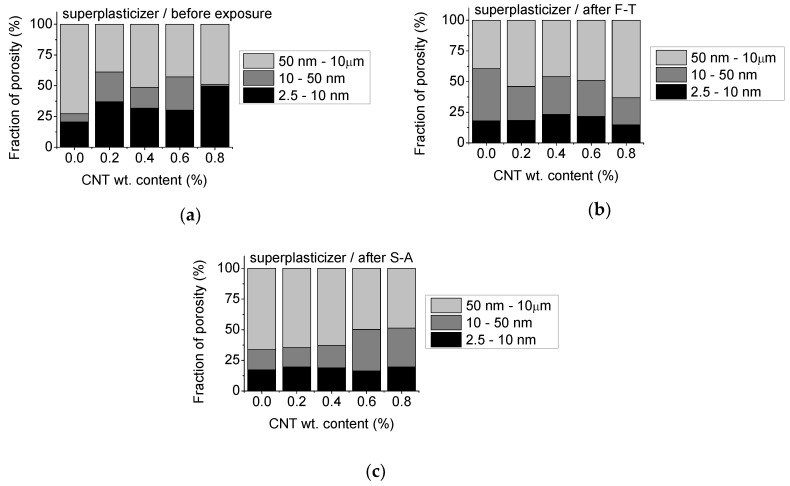
Fraction of gel (2.5–10 nm), medium capillary (10–50 nm) and large capillary (50 nm–10 μm) pores of mortars prepared using superplasticizer as dispersant: (**a**) before exposure, (**b**) after F-T and (**c**) after S-A.

**Figure 8:**
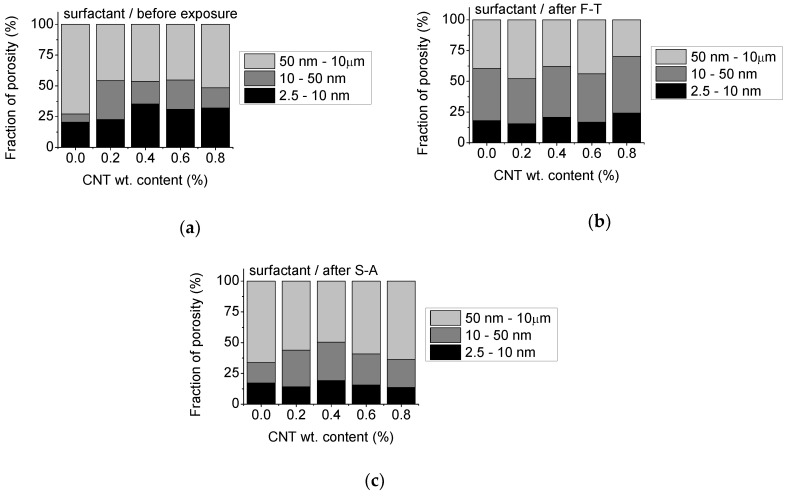
Fraction of gel (2.5–10 nm), medium capillary (10–50 nm) and large capillary (50 nm–10 μm) pores of mortars prepared using superplasticizer as dispersant: (**a**) before exposure, (**b**) after F-T and (**c**) after S-A.

Since transport is favored through capillary pores the fraction of porosity corresponding to specific pores sizes appears highly relevant for prediction of the durability of cementitious materials [22,23,29,35,36,37]. Depending on their size, pores have been categorized as either gel pores (2.5–10 nm), medium capillary pores (10–50 nm) and large capillary pores (50 nm to 10 μm) [22,29,36]. Based on this categorization, the fraction of porosity at specific pore ranges was evaluated for the un-modified and CNT-modified mortars prepared with superplasticizer and surfactant as dispersants, respectively. The results are presented in Figure 7 and Figure 8.

By observation of the porosity data, it becomes apparent that the fraction of gel pores is higher after nano-modification, with up to 140% enhancement at 0.8 wt.% CNT content. The fraction of medium-sized capillary pores is also higher after CNT addition, except for the systems with 0.8 wt.% CNTs, where no such regime is apparent. The fraction of large-sized capillary pores is significantly lower after nano-modification in the un-exposed state (Figure 7a). Based on the data presented in Figure 7b, gel pores present a 10% reduction, while medium-sized capillary pores are considerably enhanced after F-T in the un-modified specimens. Similarly, nano-modified specimens present a drop in the fraction of gel pores and a respective increase of their medium-sized capillaries after F-T, which is more evident at higher nano-modification concentrations (0.8 wt.%). At the same time, S-A deteriorates the microstructure of both the un-modified and the CNT-modified specimens with up to 60% drop of the fraction of gel pores (Figure 7c). As the CNT content increases medium-sized pores are developed over large-sized ones after S-A. Nevertheless, large size capillaries still dominate over medium-sized ones after F-T and S-A exposure in nano-modified specimens prepared with superplasticizer.

Before exposure, nano-modified specimens prepared with surfactant (Figure 8a) show similar trends with those prepared with superplasticizer (Figure 7a), i.e., increased fraction of gel and medium-sized pores after nano-modification and up to 38% drop of the large-sized ones. As observed in Figure 8b, F-T results in a reduction of the fraction of the gel pores compared to the un-exposed state, and a respective increase of the medium sized ones, while large-sized pores present a variation with the CNT content. Finally, as demonstrated in Figure 8c, the fraction of large sized capillary pores is higher after S-A exposure, while gel pores are considerably reduced in surfactant-based nano-modified mortars.

### 3.2. Transport Properties

**Figure 9:**
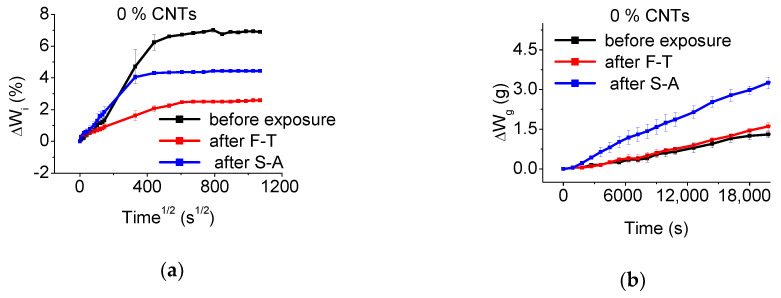
Effect of exposure state on the (**a**) water absorption, and (**b**) gas permeability profiles of un-modified mortars**.**

**Figure 10:**
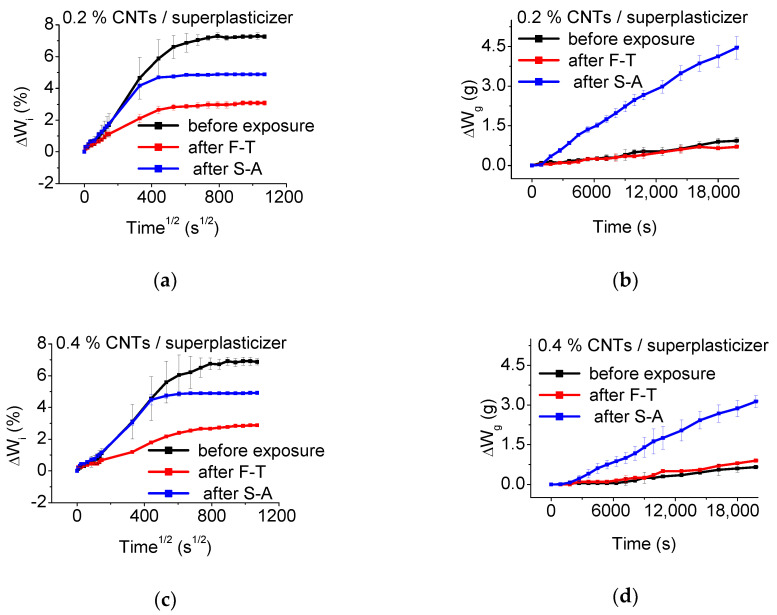

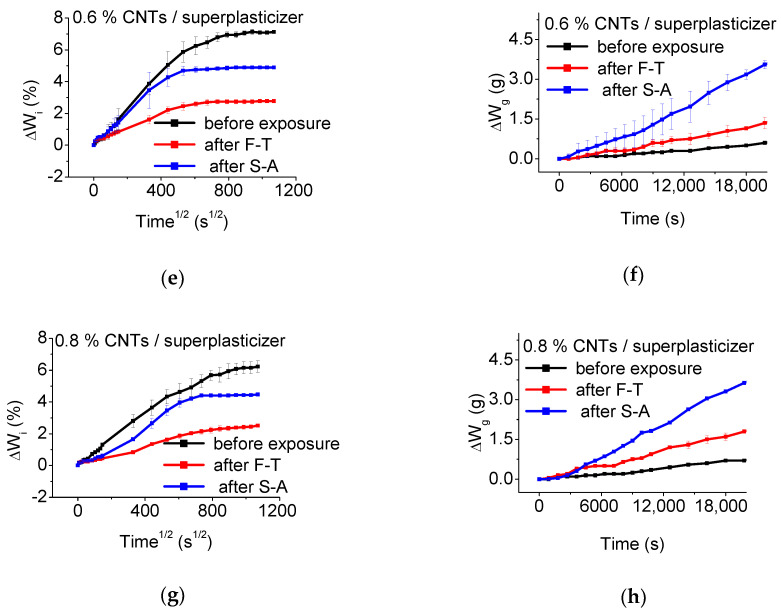
Effect of exposure state on the water absorption (**a**,**c**,**e**,**g**) and gas permeability (**b**,**d**,**f**,**h**) profiles of mortars with: (**a**,**b**) 0.2, (**c**,**d**) 0.4, (**e**,**f**) 0.6, and (**g**,**h**) 0.8 wt.% CNT contents, prepared using superplasticizer as dispersant.

**Figure 11:**
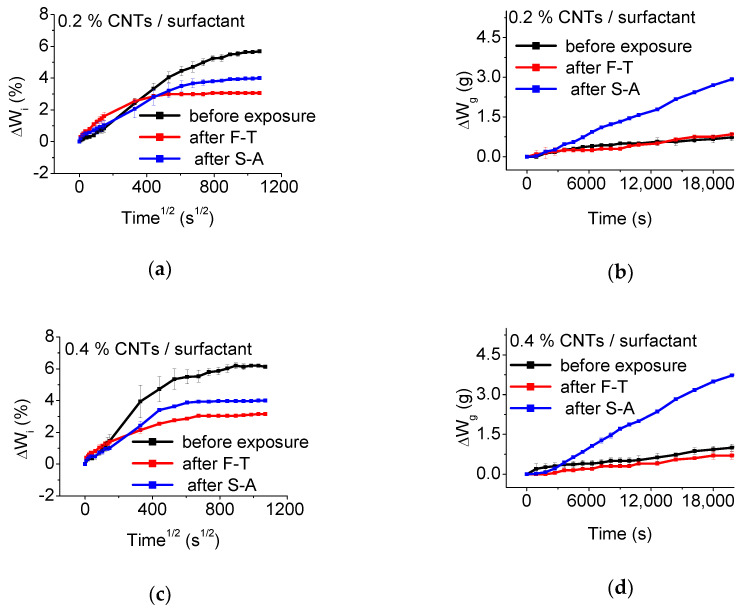

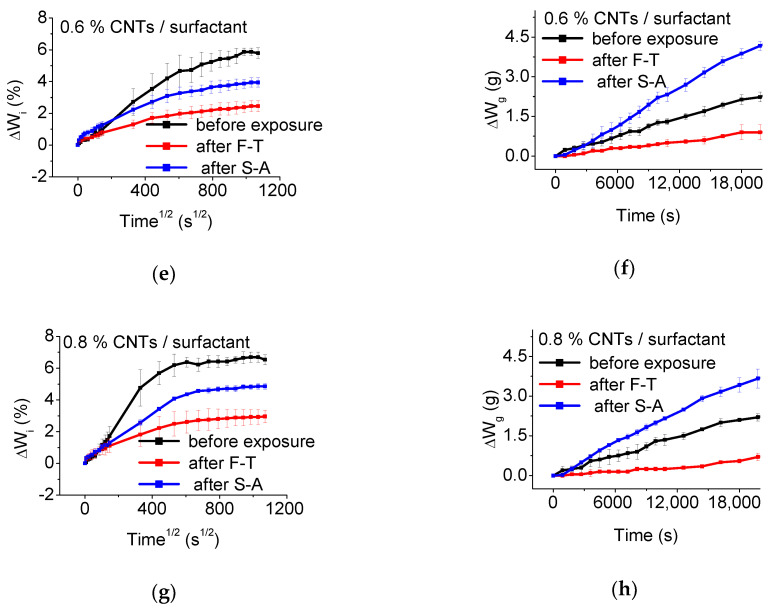
Effect of exposure state on the water absorption (**a**,**c**,**e**,**g**) and gas permeability (**b**,**d**,**f**,**h**) profiles of mortars with: (**a**,**b**) 0.2, (**c**,**d**) 0.4, (**e**,**f**) 0.6, and (**g**,**h**) 0.8 wt.% CNT contents, prepared using surfactant as dispersant.

The water absorption- and gas permeability- profiles, before and after exposure, of the un-modified and CNT-modified mortars prepared with superplasticizer and surfactant are depicted in Figure 9, Figure 10 and Figure 11, respectively. The calculated Dm and ΔW_s_ of the un-modified and CNT-modified mortars are included in Table 1, while the respective ΔŴ_g_ values are presented in Table 2, before and after exposure.

As observed in Figure 9a and Table 1, while at very early stages of exposure the un-modified specimens present similar water absorption profiles, their diffusion coefficient is slightly reduced after exposure to F-T cycling, while S-A results in app. 65% higher diffusion rates. Interestingly, F-T and S-A exposure results in app. 63% and 36% reduction of the ΔWs of the un-modified mortars. One plausible explanation of the observed behavior could be the reduced amounts of pores after F-T and S-A exposure due to continuation of hydration during exposure (see Figure 3). It is however interesting to note that the amount of porosity of plain mortars after F-T and S-A is almost the same, while their Dm and ΔWs values differ drastically. Based on the porosity analysis (Figure 7), although un-modified mortars contain the same level of capillary pores after F-T and S-A exposure, the fraction of large-sized capillaries is lower after F-T. Thus, it can be deduced that water diffuses faster through and can be easier contained in the larger sized capillaries of S-A specimens.

At the same time, the results presented in Figure 9b reveal that F-T leads in slight deterioration of the gas permeability profile of the un-modified specimens with app. 18% higher ΔŴg, while methanol permeates much easier through plain mortars after S-A presenting app. 162% higher loss compared to the un-exposed state (see Table 2). These results cannot be directly explained based on the porosity data since the total porosity of the un-modified samples is reduced after the environmental exposure (Figure 3). At the same time, the number of capillary pores is slightly increased after environmental exposure, without however significant difference between F-T and S-A state. As in the case of the water absorption results, the big difference in the gas permeability after S-A could be associated with the higher number of large capillaries, along with structural changes that cannot be captured by the porosimeter data (resolution of up to 10 μm).

As observed in Figure 10a,c,e,g, the absorption profiles of the CNT-modified specimens prepared with superplasticizer present similarities among different CNT contents, as well as with the curves of the un-modified mortars. These can be summarized in that exposure results in considerable reduction of the absorbed water after F-T and S-A. However, a detailed analysis of the absorption results, presented in Table 2, reveal that nano-modified mortars present a great variation of their D_m_ and ΔW_s_ values as a function of CNT content and exposure state. D_m_ does not show a monotonic variation after F-T and S-A, while ΔW_s_ values are clearly reduced. Nano-modification is beneficial as it results in reduced water diffusion both in the un-exposed (up to app. 65%) and the exposed state (up to app. 80%). The higher the amount of CNTs, the higher the reduction of the Dm before and after exposure. The beneficial role of nano-modification is however not observed in the ΔW_s_ values of superplasticizer-based specimens since they are similar or slightly higher after CNT addition and only high levels, i.e., 0.8 wt.%, have a positive impact on the ΔWs before and after exposure (see Table 2).

The gas permeability results illustrated in Figure 10b,d,f,h indicate similar profiles between the un-modified and the nano-modified specimens before and after F-T exposure for up to 0.4 wt.% CNT contents. The addition of CNTs using superplasticizer is beneficial for the gas permeability of the mortars before exposure, as the ΔŴ_g_ values drop from 0.23 g/h to as low as 0.10 g/h (app. 56%) upon addition of 0.6 wt.% CNTs (see Table 3). ΔŴ_g_ values of nano-modified specimens remain overall lower than the respective ones of un-modified specimens after F-T. However, as the CNT content increases, permeability also increases, and at 0.8 wt.% CNTs a deterioration in the ΔŴ_g_ values is observed after F-T (see Table 3). Finally, S-A adversely impacts the permeation of the nano-modified mortars compared to the un-exposed state as indicated by the % change of the ΔŴ_g_ values. However, it is important to note that the absolute ΔŴ_g_ values of the nano-modified specimens are in most cases close to those of the un-modified ones after S-A.

As observed in Table 2, nano-modification is beneficial for the Dm values of the mortars prepared with surfactant before exposure, which demonstrate an up to 65% decrease at 0.2 wt.% CNT content. Diffusion is faster after F-T in surfactant-based nano-modified mortars, compared to the un-exposed state (see Figure 11 and Table 2). Interestingly, S-A results in lower diffusion rates after nano-modification and compared to the un-exposed state. As in the case of superplasticizer-based specimens, nano-modified mortars with surfactant show lower levels of plateau values after F-T and S-A exposure.

The gas transport properties of the nano-modified mortars prepared with surfactant present similarities with the respective properties of the un-modified ones and those prepared with superplasticizer, at low CNT levels (up to 0.4 wt.% CNT contents), as observed in Figure 11b,d. Interestingly, after F-T the gas permeability curves of the surfactant-based specimens with 0.6 and 0.8 wt.% CNTs are below those of the un-exposed state (see Figure 11f,h). Based on the ΔŴ_g_ values presented in Table 3, this is mainly associated with the great increase of the gas permeability after the addition of a high amount of CNTs in the un-exposed state and the very low ΔŴ_g_ values after F-T of all surfactant-based nano-modified specimens. Finally, nano-modification, aided by surfactant, results overall in higher ΔŴ_g_ values compared with the plain specimens. It is however important to note that the % ΔŴ_g_ change due to S-A is considerably lower in surfactant-based specimens compared with those prepared with superplasticizer as dispersant.

## 4. Discussion

Based on the porosity data, mortars present overall an increase in their capillary porosity after F-T and S-A. The frost action during F-T results in interconnection of the pores while the high pressure developed due to phase transitions (formation of ettringite and/or gypsum) in the confined space of pores tends to widen their magnitude during S-A. The main differences in the microstructure after F-T as a function of dispersant is that superplasticizer-based mortars exhibit an increase of both medium-sized and large capillary pores, while surfactant-aided nano-modified mortars show mainly higher amounts of medium-sized capillaries. This behavior in superplasticizer-based specimens can be linked with the presence of CNT-agglomerates which act as stress concentrators resulting in debonding at the nanoscale. The slightly better response of surfactant-based specimens can be thus attributed to better dispersed and individual CNTs. It is however important to note that nano-modification is overall beneficial, since the fraction of capillary pores (medium and large sized) of nano-modified specimens after exposure remains in most cases equal or slightly lower compared to the one of the un-modified specimens. The only exemption is found after S-A exposure of surfactant-based specimens with 0.8 wt.% CNTs, which show slightly higher fraction of capillary pores.

As observed in the previous paragraphs the variation of the transport properties is not monotonic with the CNT content, type of dispersant, and exposure state. Based on the water transport data, it can be assumed that the refinement of the pores through nano-modification delays the diffusion of water. However, water is not only contained in the free volume, but also through interaction mechanisms in cementitious media. As discussed previously, the poly-carboxylate nature of the superplasticizer used as dispersant is susceptible to water through interaction of its polar groups with water [29]. This may explain the observed variations in the ΔWs values as a function of CNT/ superplasticizer content, in superplasticizer-based mortars. Furthermore, it is difficult to correlate the obtained response with key porosity data, like the amount/fraction of gel/capillary pores, and/or the total porosity of the specimens. The main reason is that not only the nano-inclusions but also the type of dispersants influences the environmental response of the nano-modified mortars. For instance, surfactants may act as air-entraining agents [38]. This hinders the creation of micro/macro-cracks during F-T of the mortars and may explains the lower gas permeability of surfactant-based specimens after F-T compared to the un-modified and CNT-modified ones prepared with superplasticizer, which are highly compacted as the amount of CNT/superplasticizer increases. At the same time, the poly-carboxyl nature of the superplasticizer used in the current study has been connected in the past with increased damage due to S-A. This has been linked with the absorption of the poly-carboxylate material by the sulfate products and thus, reduced cohesion of the cementitious matrix [39,40]. According to Marchon, et al. [41] one way to reduce the sulfate sensitivity of poly-carboxylate superplasticizers is by introducing high anionic molecules. Based on the above, the anionic nature of the surfactant used in the current study should have led to a superior response of surfactant-based nano-mortars over superplasticizer-based ones after S-A. This has only been observed in terms of water absorption and not gas transport properties. Thus, apart from the pore changes, it is obvious that macrostructural changes and possibly interactions between the absorption medium and the dispersants should be taken into account in order to evaluate the transport properties of the nano-modified mortars before and after exposure.

## 5. Conclusions

The current study investigates the effect of F-T and S-A exposure on the pore structure and transport properties of CNT-modified mortars. Key factors under investigation are the CNT content and the type of dispersant (superplasticizer versus surfactant). Based on the obtained results, the following conclusions can be drawn:

Pore structure:

- CNT inclusion results in a considerable drop of the total porosity and a respective enhancement of the amount of the gel and medium-sized pores in the un-exposed state.
- F-T and S-A lead to a slight reduction of the fraction of gel pores and an enhancement of medium sized ones before nano-modification.
- Nano-modified specimens present a substantial decrease of the fraction of gel pores after F-T and S-A exposure. Large-sized capillaries dominate over medium-sized ones when superplasticizer is used as dispersant. Surfactant-based mortars follow this trend after S-A, while after F-T, medium-sized capillaries dominate.

Transport properties

- Water absorption is reduced in both un-modified and nano-modified mortars after F-T and S-A. Nano-modification is beneficial for the diffusion coefficient however the total amount of absorbed water is overall slightly higher in CNT-containing specimens both in the un-exposed and the exposed state. The only exception is observed in surfactant-based specimens after S-A.
- Gas permeability is worsened after F-T and S-A in un-modified and CNT-modified specimens with superplasticizer. Surfactant-based specimens show an improvement after F-T with very low gas flow rate values compared to the un-exposed state.
- Transport properties cannot be directly connected with the microstructural properties expressed by the porosity of the prepared specimens. Both the presence of nano-fillers and the nature of the dispersant influence the resultant properties.

Overall, it can be concluded that it is not possible to predict the durability of CNT-modified cementitious materials based on the pore structure data before exposure. Apart from the role of CNTs in filling the pores in the un-exposed state, one should consider the role of dispersants on the microstructure and durability before and after exposure. Thus, the durability aspect of nano-modified system is quite complicated and needs to be assessed as part of the evaluation process of these new materials. Moreover, a complete study on the interaction of CNTs, dispersants and key exposure conditions, including a chemical analysis of the degradation products, could provide greater insight for the design of CNT-modified cementitious materials.

## Figures and Tables

**Table 1:** Quantities of all admixtures used for the preparation of each composition.

CNT Content %	CNT Content g	Dispersive Medium	Dispersant g	Anti-Foaming Agent g	Workability Modifier g
0	0	-	0	0	1.5
0.2	0.9	Superpl.	1.4	0	1.7
0.4	1.8		2.7	0	0.9
0.6	2.7		4.1	0	0.5
0.8	3.6		6.8	0	0.1
0.2	0.9	Surf.	0.5	0.7	0.9
0.4	1.8		0.9	0.7	1.8
0.6	2.7		1.4	0.7	1.9
0.8	3.6		1.8	0.7	2.1

**Table 2:** Diffusion coefficient, D_m_ and relative weight gain at saturation, ΔW_s_, as a function of CNT content, dispersive medium and exposure state.

CNT Content %	Dispersive Medium	D_m_ B-E ^1^ 10^−3^ mm^2^/s	ΔW_s_ B-E ^1^ %	D_m_ F-T ^1^ 10^−3^ mm^2^/s	ΔW_s_ F-T ^1^ %	D_m_ S-A ^1^ 10^−3^ mm^2^/s	ΔW_s_ S-A ^1^ %
0	-	1.62 ± 0.07	6.91 ± 0.03	1.22 ± 0.02	2.57 ± 0.03	2.66 ± 0.05	4.44 ± 0.00
0.,2	Superpl.	1.08 ± 0.03	7.25 ± 0.03	1.18 ± 0.02	3.08 ± 0.00	2.51 ± 0.05	4.88 ± 0.00
0.4		0.80 ± 0.04	6.89 ± 0.04	0.30 ± 0.01	2.87 ± 0.03	1.28 ± 0.03	4.91 ± 0.02
0.6		0.58 ± 0.03	7.10 ± 0.03	0.77 ± 0.02	2.77 ± 0.02	0.95 ± 0.02	4.89 ± 0.00
0.8		0.58 ± 0.04	6.11 ± 0.11	0.22 ± 0.00	2.45 ± 0.06	0.51 ± 0.01	4.45 ± 0.02
0,2	Surf.	0.57 ±0.04	5.60 ± 0.08	2.87 ± 0.06	3.42 ± 0.00	0.69 ± 0.01	3.97 ± 0.03
0,4		0.71 ± 0.05	6.17 ± 0.04	1.75 ± 0.04	3.13 ± 0.03	0.62 ± 0.01	3.98 ± 0.02
0,6		0.81 ± 0.02	5.72 ± 0.18	0.77 ± 0.02	2.42 ± 0.05	0.59 ± 0.01	3.91 ± 0.06
0,8		1.05 ± 0.01	6.62 ± 0.08	1.43 ± 0.03	2.93 ± 0.03	0.81 ± 0.02	4.84 ± 0.02

^1^ B-E: stands for before exposure, F-T for Freeze-Thaw and S-A for sulfate attack.

**Table 3:** Mass flow rate of methanol, ΔŴg as a function of CNT content, dispersive medium and exposure state.

CNT Content %	Dispersive Medium	ΔŴg B-E ^1^ g/h	Adj. R-Square	ΔŴg F-T ^1^ g/h	Adj. R-Square	ΔŴg S-A ^1^ g/h	Adj. R-Square
0	-	0.23	0.99	0.27	0.99	0.61	1.00
0.2	Superplast.	0.17	0.99	0.14	0.99	0.84	1.00
0.4		0.11	0.96	0.15	0.97	0.57	1.00
0.6		0.10	0.99	0.23	0.99	0.61	0.99
0.8		0.13	0.98	0.32	1.00	0.63	0.99
0.2	Surf.	0.15	0.96	0.15	0.99	0.53	1.00
0.4		0.19	0.98	0.13	0.99	0.67	1.00
0.6		0.43	1.00	0.16	1.00	0.76	1.00
0.8		0.42	1.00	0.11	0.96	0.69	1.00

^1^ B-E: stands for before exposure, F-T for Freeze-Thaw and S-A for sulfate attack.
